# Deceptively Challenging Airway Management in a CHARGE Syndrome Patient Despite Careful Preoperative Anticipation: A Case Report

**DOI:** 10.1155/carm/2029479

**Published:** 2026-08-31

**Authors:** Masoud Nashibi, Seyedeh Fatemeh Hamzavi, Sogol Asgari

**Affiliations:** ^1^ Department of Anesthesiology, School of Medicine, Anesthesiology Research Center, Loghman Hakim Hospital, Shahid Beheshti University of Medical Sciences, Tehran, Iran, sbmu.ac.ir; ^2^ Students Research Committee, School of Medicine, Shahid Beheshti University of Medical Sciences, Tehran, Iran, sbmu.ac.ir; ^3^ Hearing Disorders Research Center, Loghman Hakim Hospital, Shahid Beheshti University of Medical Sciences, Tehran, Iran, sbmu.ac.ir

**Keywords:** airway management, anesthesia, CHARGE syndrome, difficult intubation, video laryngoscopy

## Abstract

Difficult intubation is a critical challenge in anesthesia, often linked to anatomical abnormalities. CHARGE syndrome patients are known for airway difficulties due to craniofacial anomalies, yet some cases may present without obvious anatomical predictors. We report the case of an 8‐year‐old girl with CHARGE syndrome undergoing surgery for choanal atresia. Despite a history of multiple previous surgeries without reported intubation difficulties, she experienced difficult intubation despite the absence of classical anatomical predictors. Standard direct laryngoscopy attempts failed two times, necessitating the use of video laryngoscopy for successful intubation. Preoperative imaging revealed nasal obstruction and severe septal deviation but no significant craniofacial abnormalities that could predict intubation difficulty. This case highlights the unpredictable nature of difficult intubation in CHARGE syndrome, even in patients without apparent anatomical risk factors. The underlying cause remained unclear, suggesting the possibility of subtle airway anomalies or dynamic factors during intubation. The successful use of video laryngoscopy underscores its value in managing difficult airways in such cases. Clinicians should maintain a high index of suspicion for difficult airway management in CHARGE syndrome patients, even in the absence of overt anatomical abnormalities. Preoperative planning should include preparation for advanced airway techniques such as video laryngoscopy to improve patient safety and outcomes.

## 1. Introduction

Difficult intubation is defined as the failure or difficulty of securing the airway using conventional laryngoscopy, which often necessitates multiple attempts, extended intubation time, or alternative airway management techniques to ensure adequate ventilation and oxygenation [1, 2]. It is a serious concern in anesthesiology, as aspiration, hypoxia, cardiovascular arrest, and even mortality may occur if the airway is not secured promptly [1, 3]. There are many reasons that contribute to difficult intubation, including both internal and external ones. Anatomical variations, such as limited mouth opening, restricted cervical movement, airway obstruction from tumors, and prominent upper incisors, can make it difficult to visualize and pass the endotracheal tube [1]. Additionally, conditions such as obesity, craniofacial anomalies, temporomandibular joint disorders, and diseases such as rheumatoid arthritis can contribute to the challenges of intubation [2].

CHARGE syndrome is a genetic disorder characterized by a combination of congenital anomalies described by its acronym CHARGE: Coloboma, Heart defects, Atresia choanae, Restriction of growth and development, Genital abnormalities, and Ear abnormalities [1]. It is most frequently linked to mutations in the CHD7 gene, which plays a critical role in embryogenesis, particularly in craniofacial development and neural crest cell differentiation [4]. These structural abnormalities often complicate airway management, as features such as cleft palate, micrognathia, choanal atresia, and hypertrophic tonsils can make both mask ventilation and intubation challenging [5]. Furthermore, a high‐positioned larynx and potential upper airway obstruction increase the risk of difficult intubation and perioperative respiratory complications. Because children with CHARGE syndrome frequently require early surgical interventions, careful preoperative airway assessment and planning for advanced techniques, such as video laryngoscopy or fiberoptic intubation, are essential to ensure safe anesthesia and minimize complications [1, 6].

In some cases, difficult intubation can be predicted through a complete preoperative assessment that evaluates key factors, including the Mallampati classification, thyromental distance, and cervical spine mobility [2, 7]. These evaluations help identify potential airway challenges and guide the choice of appropriate airway management strategies. Advanced airway management techniques, such as video laryngoscopy, fiberoptic intubation, or the use of supraglottic airway devices, are often essential in these situations [8, 9]. Video laryngoscopy is a highly effective alternative for managing difficult airways. Its use is particularly valuable in situations where the airway is deceptively challenging, as may occur in pediatric syndromic patients without obvious anatomical anomalies. Unlike direct laryngoscopy, which requires a clear view of the vocal cords, it uses a camera on the blade to provide a magnified view of the airway [10]. This improves glottic accessibility, increases first‐attempt success rates, and reduces airway trauma, especially in patients with limited neck mobility or anatomical abnormalities [8]. It also enhances teamwork, as multiple providers can see the airway on‐screen and assist in real time. Because of these benefits, video laryngoscopy is now widely recommended as a first‐line or backup method for difficult intubation [8, 11]. This is especially relevant in syndromic patients such as those with CHARGE, where anatomical anomalies may not be externally apparent but significantly impair airway access.

This case report aims to illustrate a deceptively challenging airway in a patient with CHARGE syndrome, review the management strategies employed, and provide lessons to improve preparation and outcomes in similar high‐risk pediatric patients.

## 2. Case

An 8‐year‐old girl with CHARGE syndrome was admitted for surgical management of choanal atresia. She presented with purulent nasal discharge and right‐sided nasal obstruction. Although her family history was unremarkable and her allergy history was insignificant, her past medical history included surgical repair of cleft lip and palate, bilateral congenital hydrocephalus, and progressive bilateral sensorineural hearing loss. She had also undergone endovascular closure of a patent ductus arteriosus (PDA) and ventilation tube (VT) placement at the age of 2. However, despite undergoing multiple previous surgeries, there was no reported history of difficult intubation during these procedures.

**FIGURE 1:**
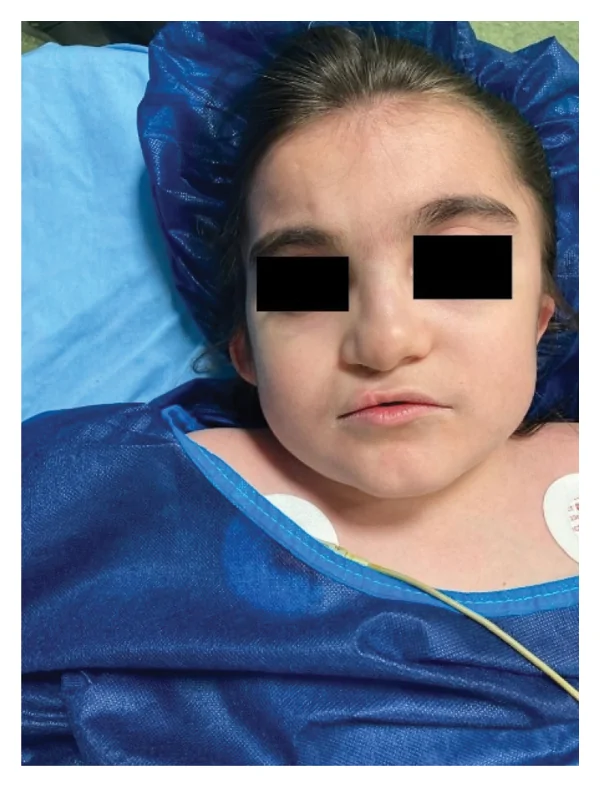
Dysmorphic facial features and cleft lip surgical scar in the patient.

**FIGURE 2:**
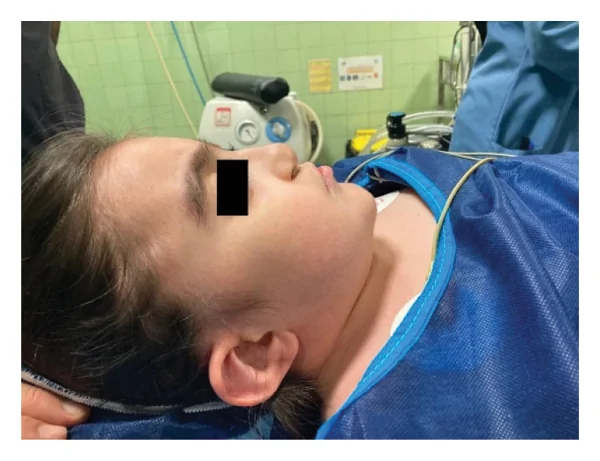
Lateral view of the patient’s neck.

On physical examination, the patient had dysmorphic facial features consistent with her syndrome, and a surgical scar from the cleft lip repair was visible (Figures 1 and 2). No masses were observed on anterior rhinoscopy. Nasopharyngoscopy was considered to assess the upper airway but not performed due to the patient’s young age, lack of cooperation, and the unavailability of proper sedative or anesthetic agents in our resource‐limited setting. Under optimal conditions and in the hands of experienced personnel, this examination can be safely accomplished in children, including those with craniofacial anomalies, by applying supportive approaches such as mild sedation or hypnosis while preserving spontaneous ventilation. Therefore, cone‐beam computed tomography (CBCT) was chosen as an alternative and reviewed preoperatively to visualize the upper airway, characterize the degree of nasal obstruction and secretion burden, and exclude space‐occupying lesions that could further complicate ventilation or laryngoscopy.

**FIGURE 3:**
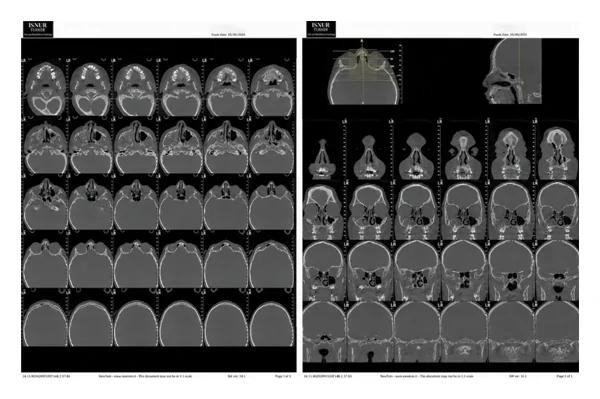
CBCT images of the paranasal sinuses in axial and coronal planes.

CBCT imaging showed significant mucosal secretions and a bony plate obstructing the right choana, along with severe nasal septal deviation attaching to the right inferior turbinate (Figure 3). Additionally, there was marked right inferior turbinate hypertrophy, complete opacification of the right maxillary sinus, and absence of the right maxillary lateral incisor, leading to localized bone loss. No space‐occupying lesions were detected, and while the right osteomeatal complex (OMC) was entirely obstructed, the left OMC remained patent.

Given the patient’s syndromic condition, we performed a focused preoperative airway assessment. Available prior anesthetic records were reviewed and did not document difficult mask ventilation or tracheal intubation. Bedside evaluation included mouth opening, dentition, mandibular size, and cervical spine mobility; a formal Mallampati score and cooperative measures (e.g., thyromental distance) were not reliably obtainable because of limited cooperation. CBCT which was reviewed for gross craniofacial predictors of difficult intubation, showed no clear predictors other than excessive secretions. Although CBCT did not demonstrate obvious craniofacial predictors of difficult intubation, we considered the diagnosis of CHARGE syndrome, the history of multiple prior surgeries, and the dysmorphic facial appearance (Figures 1 and 2) as established risk factors for a potentially difficult airway. Subsequently, a stepwise airway plan was agreed between the anesthesia and ENT teams:

• Plan A: Direct laryngoscopy under optimal conditions with a single supervised attempt by a trainee and immediate takeover by the attending if unsuccessful.
• Plan B: Early switch to video laryngoscopy with bougie.
• Plan C: Supraglottic airway for rescue ventilation if needed.
• Plan D: Emergency surgical airway with ENT standby.

Before surgery, a full set of laboratory tests was conducted, all showing normal results (Table 1). Cardiology and anesthesia evaluations were performed, and the patient was cleared for surgery.

**TABLE 1:** Preoperative laboratory test results.

Test	Result	Unit	Reference value
*CBC*			
WBC	7.97	10^3^/μL	3.9–11.5
RBC	5.40	10^6^/μL	3.9–5.4
HGB	14.2	g/dL	12.8–16
HCT	43.1	%	37–48
M.C.V.	79.8	fL	77–98
PLATELETS	325	10^3^/μL	150–450
E.S.R 1^st^ h	10	mm/h	Up to 10

*Hemostasis*			
P.T.	14	Sec	—
INR	1	—	—
P.T.T.	38	Sec	—

*Blood Biochemistry*			
Urea	15	mg/dL	10–50
Creatinine	0.7	mg/dL	Child: 0.3–0.8 Male: 0.9–1.3 Female: 0.6–1.2

*Serology*			
CRP. (Titer)	2.6	—	Up to 6

During surgery, given the anticipated difficult intubation and the patient’s young age and lack of cooperation for an awake fiberoptic approach, general anesthesia (GA) was induced using rapid sequence induction (RSI). The patient received 2 mg/kg of propofol and 1 mg/kg of succinylcholine. Advanced airway equipment, including a video laryngoscope and bougie, was prepared in advance and kept immediately available as the planned backup due to the patient’s syndromic background.

**FIGURE 4:**
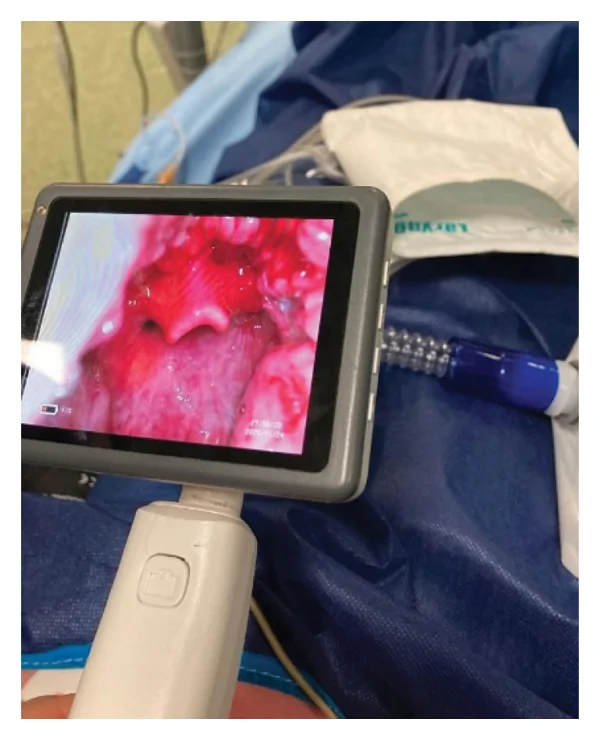
Successful intubation using video laryngoscopy after failed direct laryngoscopy attempts.

Following the onset of fasciculations, intubation was attempted. Consistent with our preoperative airway plan, the team agreed to limit direct laryngoscopy attempts and transition early to video laryngoscopy if adequate glottic visualization was not achieved. The first direct laryngoscopy attempt was performed by a resident but was unsuccessful. A second attempt was then performed by the attending anesthesiologist; however, adequate glottic visualization could not be achieved. To avoid repeated laryngoscopy attempts, the team promptly transitioned to video laryngoscopy. Eventually, the patient was successfully intubated using video laryngoscopy (Figure 4).

The cause of the difficult intubation remained unclear. The patient had no anatomical abnormalities in the neck, such as micrognathia, retrognathia, or cervical spine limitations, nor any oropharyngeal masses, macroglossia, or laryngeal anomalies that could explain the difficulty. Despite a history of cardiac surgery, there was no evidence of recurrent laryngeal nerve injury, which is more commonly linked to postoperative airway issues rather than failed intubation. With no clear reason, the difficulty with intubation remained unexplained.

## 3. Discussion

In this case report, we present an 8‐year‐old girl with CHARGE syndrome who underwent surgery for choanal atresia. Although the patient was recognized preoperatively as being at increased risk for airway difficulty due to her underlying syndromic condition and facial features (Figures 1 and 2), no classical anatomical predictors of severe intubation difficulty were identified, and she had previously undergone multiple anesthetics without documented airway complications. Therefore, the airway was managed as potentially high risk from the outset, with advanced devices prepared and a stepwise escalation plan agreed with ENT backup. Nevertheless, supervised attempts at direct laryngoscopy were unsuccessful, and tracheal intubation was ultimately achieved using video laryngoscopy.

This case is significant because it highlights the limitations of conventional preoperative airway assessments, which primarily focus on structural abnormalities. However, as seen here, in syndromic patients such as our patient, functional or dynamic airway factors may play an equally important role in airway difficulty and may not be apparent during routine bedside evaluation. Recognizing these limitations emphasizes the need for a more comprehensive approach to airway evaluation and reinforces the importance of having advanced intubation techniques, such as video laryngoscopy, readily available.

CHARGE syndrome, a complex genetic disorder affecting multiple systems, often presents with craniofacial anomalies and a spectrum of airway abnormalities that can complicate intubation [12]. While some individuals exhibit clear structural issues like choanal atresia, cranial nerve IX and X dysfunction, or laryngomalacia, as detailed in Table 2, others, like our patient, may have subtle functional challenges not detected by routine assessments [5, 6, 12, 13]. These functional issues, including neuromuscular dysfunction, soft tissue collapsibility, or atypical airway reflexes, can contribute to unanticipated intubation difficulties, such as poor glottic visualization, increased risk of airway obstruction, or challenges in advancing the endotracheal tube [6, 14]. Consequently, conventional screening methods, which primarily assess structural features, may fail to detect these functional contributors, thereby explaining why some patients encounter greater‐than‐expected intubation difficulties despite apparently normal anatomical findings.

**TABLE 2:** Airway anomalies in CHARGE syndrome and their impact on intubation.

Anomaly	Description	Prevalence in CHARGE syndrome	Impact on airway and intubation
Choanal Atresia/Stenosis [5]	Narrowing or blockage of nasal passages by bone or soft tissue.	50%–60%	Obstructs nasotracheal intubation; complicates nasopharyngoscopy; may cause respiratory distress.
Micrognathia [13]	Abnormally small jaw, reducing oral cavity space.	Common (∼43%)	Reduces space for laryngoscopy; complicates visualization of vocal cords.
Laryngeal Clefts [13]	Abnormal communication between larynx and esophagus due to incomplete separation.	Less common (∼32%)	Increases aspiration risk; risks tube misplacement into the esophagus.
Cleft Lip/Palate [5]	Congenital fissure in the upper lip and/or palate, affecting oral cavity structure.	20%–40%	Indirectly affects airway by altering oral cavity shape; increases aspiration risk.
Tracheobronchial Stenosis [14]	Narrowing of the trachea or bronchi, which can be congenital or acquired.	Rare	Significantly complicates intubation and ventilation; may require specialized equipment.
Subglottic Stenosis [15]	Narrowing of the airway below the vocal cords, often congenital.	Variable (2%–20%)	Hinders passage of endotracheal tube; increases risk of post‐extubation obstruction.
Vocal Cord Paralysis [14]	Impaired vocal cord movement due to cranial nerve dysfunction (e.g., vagus nerve).	Common	Obstructs glottic view during laryngoscopy; may cause postoperative airway issues.
Nasal Obstruction [14]	Blockage of the nasal cavity due to anatomical variations like septal deviation or turbinate hypertrophy.	Common	Prevents nasotracheal intubation; may indicate other airway challenges.
Laryngeal Hypotonia [14]	Reduced muscle tone in the larynx, a functional anomaly.	Variable	Causes unexpected airway collapse during intubation; often not detectable preoperatively.
Laryngomalacia [16]	Softness and inward collapse of supraglottic structures during inspiration, leading to airway obstruction.	Common in infants	Causes dynamic airway obstruction; may complicate tube placement.
Tracheoesophageal Fistula (TEF) [5]	Abnormal connection between trachea and esophagus.	15%–25%	Risks ventilating stomach or aspiration during intubation.
Excessive Secretions [16]	due to salivary pooling or mucus production.	Common	Impairs visualization and increases aspiration risk during intubation.
Cranial Nerve Abnormalities [5]	Dysfunction of glossopharyngeal (IX) and vagus (X) nerves, affecting airway reflexes.	IX/X: 70%–80%	Impairs swallowing and airway protective reflexes, increasing aspiration risk; complicates diagnostic procedures.

Our patient’s difficult intubation, despite prior uneventful intubations, raises the question of whether the challenge was CHARGE syndrome‐related or situational. CHARGE syndrome can involve static anomalies like choanal atresia or functional issues like laryngeal hypotonia [5, 13, 14]. However, preoperative CBCT showed no structural issues predicting difficulty, only excessive secretions. These secretions, common in CHARGE syndrome [16], likely obscured glottic visualization during direct laryngoscopy. Furthermore, RSI with succinylcholine may have exacerbated dynamic airway collapse, possibly due to laryngeal hypotonia [14]. Thus, this suggests a situational challenge rather than a consistent syndrome feature. Accordingly, further research on dynamic airway factors in CHARGE syndrome is needed to improve risk assessment. Consequently, this underscores the need for advanced airway tools like video laryngoscopy, as per American Society of Anesthesiologists (ASA) guidelines [1].

In light of such situational and functional airway challenges, recent recommendations emphasize the importance of having advanced and alternative airway tools, including video laryngoscopy, available in operating rooms [1, 3, 8]. This aligns with the ASA guidelines on difficult airway management, which support early use of video laryngoscopy when initial attempts at direct laryngoscopy fail and avoid repeating intubation attempts, particularly in pediatric and syndromic patients [1]. Research has shown that compared to standard laryngoscopy, video laryngoscopy improves visualization of the vocal cords, increases the likelihood of first‐pass success, and reduces airway trauma [17]. Recent studies advocate for its routine use, particularly in pediatric cases and in patients with syndromic conditions where airway difficulties may not be apparent preoperatively [18–20].

From a management standpoint, although current difficult airway guidelines recommend early transition to alternative devices after a failed attempt [1], airway management in our teaching hospital was conducted in a supervised and structured manner. An initial attempt was performed by a resident, followed by a single attempt by the attending anesthesiologist. Advanced airway devices had been prepared in advance, and after these two unsuccessful direct laryngoscopies, no further attempts were made; the team immediately transitioned to video laryngoscopy. This limited and supervised sequence avoided repeated laryngoscopy while maintaining patient safety within an educational setting. Nevertheless, we acknowledge that in a potentially high‐risk airway, primary video laryngoscopy, or at least an earlier transition to video laryngoscopy after the first failed attempt may further reduce risk; this point has been added as a critical reflection and learning message.

This case underscores the need for a more comprehensive approach to airway evaluation. Instead of relying solely on traditional bedside assessments, anesthesiologists should consider additional strategies such as airway ultrasound, 3D imaging, or preoperative airway simulations to better assess potential risks [1]. These advanced assessments may be particularly useful in patients with syndromic craniofacial disorders (e.g., CHARGE, Pierre Robin, or Treacher Collins syndrome), previous head and neck radiotherapy, or a history of difficult direct laryngoscopy despite otherwise reassuring airway examination. Furthermore, having alternative airway management techniques readily available can enhance preparedness for unexpected intubation challenges, ensuring safer outcomes for patients.

This case demonstrates that in syndromic pediatric patients, difficult intubation may occur even in the absence of classical anatomical predictors on routine examination or imaging. It highlights the limitations of conventional airway assessment tools and reinforces the need for more advanced diagnostic methods. Given the strong evidence supporting video laryngoscopy as an effective strategy in managing airway challenges, its routine use should be encouraged, especially in pediatric and syndromic patients. Further investigation is needed to better understand the underlying mechanisms of difficult intubation in patients without obvious anatomical abnormalities, ultimately improving airway management and patient safety.

## Author Contributions

This study was designed by S.A. The experiments were performed by S.F.H. and. S.F.H. had a primary role in preparing the manuscript, which was edited by S.F.H. and M.N.

## Funding

No funding was received for this research.

## Disclosure

All authors have approved the final version of the manuscript and agree to be accountable for all aspects of the work.

## Ethics Statement

The oversight of this study was granted by the ethics committee of Shahid Beheshti University of Medical Sciences. Throughout the study, the protocol adhered strictly to the ethical guidelines outlined in the Declaration of Helsinki. The patient and her guardians in the study were thoroughly informed about the purpose and objectives of the research. Subsequently, written consent was obtained from each participant.

## Conflicts of Interest

The authors declare no conflicts of interest.

## Data Availability

The data of the case is available via enquiries from corresponding author.
